# Structural, functional and neurochemical imaging mapping of non-motor symptoms in Parkinson’s disease

**DOI:** 10.1093/braincomms/fcag316

**Published:** 2026-08-18

**Authors:** Chiara Camastra, Aldo Quattrone, Andrea Quattrone

**Affiliations:** Neuroscience Research Center, Magna Graecia University, Catanzaro 88100, Italy; Brain Health Imaging Centre, Centre for Addiction and Mental Health, University of Toronto, M5T1R8 Toronto, Canada; Neuroscience Research Center, Magna Graecia University, Catanzaro 88100, Italy; Neuroscience Research Center, Magna Graecia University, Catanzaro 88100, Italy; Institute of Neurology, Department of Medical and Surgical Sciences, Magna Graecia University, 88100 Catanzaro, Italy

**Keywords:** Parkinson’s disease, rapid eye movement sleep behaviour disorder, depression, anxiety, coordinate-based network mapping

## Abstract

Non-motor symptoms, including rapid eye movement sleep behaviour disorder (RBD), depression and anxiety, are common and often co-occurring in patients with Parkinson’s disease. This study aimed to investigate their potential shared neurobiological substrates by integrating structural, functional and neurochemical imaging data. We analysed data from 638 Parkinson’s disease patients from the Parkinson’s Progression Markers Initiative (PPMI), with available 3T T1-weighted MRI scans. RBD, depression and anxiety severity were assessed using validated clinical scales (RBD Screening Questionnaire Score, Geriatric Depression Scale and State-Trait Anxiety Inventory). Voxel-based morphometry (VBM) multivariate regression analyses were performed to identify grey matter (GM) volume loss associated with each clinical symptom. All analyses were rigorously controlled for a comprehensive set of potential confounders, including age, sex, education, disease duration, motor severity and cognitive dysfunction, thereby minimizing confounding effects related to other aspects of the disease. Coordinate-based network mapping was then applied using a large normative resting-state functional connectome (*N* = 1000), to characterize symptom-specific functional networks based on brain areas functionally connected to the VBM-derived clusters. Finally, spatial correlations between these networks and normative neurotransmitter density maps from PET data were assessed. VBM analyses revealed distinct patterns of GM atrophy across the three symptoms (pFWE<0.05), overlapping in the left middle temporal and right middle frontal gyri. The coordinate-based functional network mapping approach demonstrated that the GM atrophy pattern associated with each symptom (pFWE < 10^−6^) converged onto brain networks involving several cortical regions and overlapping across symptoms, and with the greatest spatial affinity, among canonical large-scale networks, with the Dorsal and Ventral Attention networks. All three symptom-related networks showed significant alignment with the noradrenaline transporters (NAT) spatial distribution (pFDR < 0.05). Overall, this study proposes a novel conceptual and methodological framework integrating well-established and validated techniques to identify the neuroanatomical bases of specific diseases or symptoms, potentially of interest for future research. Our neuroimaging findings in the large PPMI cohort of early Parkinson’s disease patients demonstrate that the brain networks associated with RBD, depression and anxiety non-motor symptoms were largely overlapping, involved the attention networks and were spatially aligned with the noradrenergic system, suggesting that these symptoms may have shared neurobiological substrates.

## Teaser

Camastra *et al*. report that rapid eye movement sleep behaviour disorder, depression and anxiety in Parkinson’s disease show converging structural changes that map onto shared attention networks. They further demonstrate that these networks align with the noradrenergic system, suggesting a common biological basis for these non-motor symptoms in Parkinson’s disease.

## Graphical Abstract

**Graphical Abstract:**
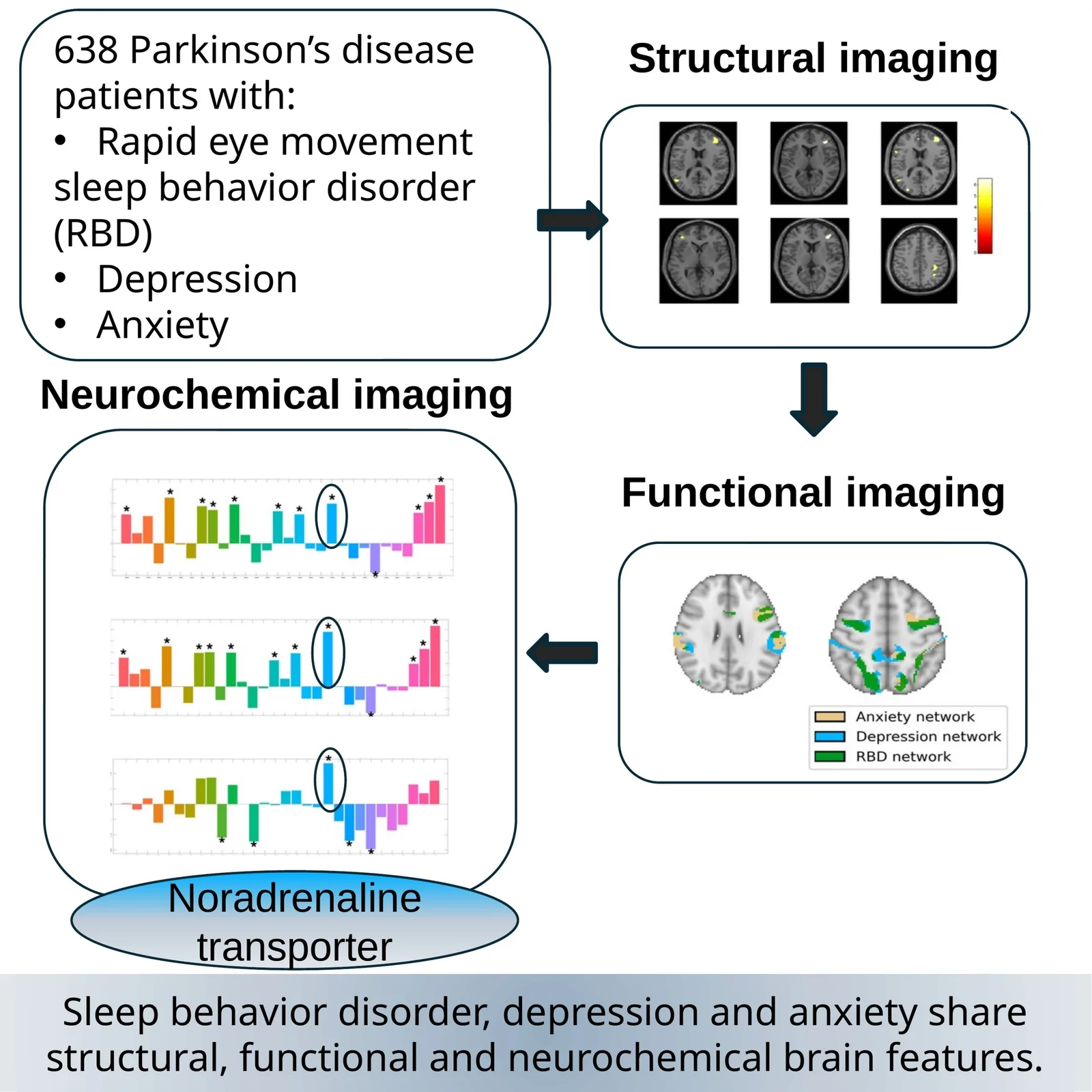

## Introduction

Parkinson’s disease is a progressive neurodegenerative disorder traditionally recognized for its core motor symptoms, including tremor, rigidity, bradykinesia and postural instability.^1^ However, it is now well established that Parkinson’s disease also encompasses a broad spectrum of non-motor symptoms, which can precede motor manifestations or appear during the disease course.^2^ Key non-motor symptoms characterizing the early Parkinson’s disease stage include rapid eye movement (REM) sleep behaviour disorder (RBD) and psychiatric features such as depression and anxiety.^3^ RBD is characterized by dream-enacting behaviours due to the loss of muscle atonia during REM sleep and is considered a strong predictor of future neurodegeneration.^4^ Depression in Parkinson’s disease often manifests with symptoms such as anhedonia, low mood and fatigue,^5^ and is commonly associated with anxiety, all of which contribute to reduced quality of life and impaired daily functioning.^6^ These non-motor symptoms frequently co-occur in patients with Parkinson’s disease, suggesting overlapping neurobiological mechanisms and a potential shared vulnerability.^7^

Recent neuroimaging studies have started to elucidate the brain mechanisms underlying these non-motor symptoms in Parkinson’s disease, with heterogeneous findings. For depression, previous data highlighted alterations within the prefrontal–limbic system and basal ganglia, suggesting a breakdown in large-scale network integration rather than damage to isolated regions.^8^ Regarding anxiety, evidence points to the involvement of the fear circuit and disruptions in the cortico–striato–thalamo–cortical limbic pathways.^9^ RBD has been associated with heterogeneous brain network dysfunction, including the Default Mode network, the Salience network and others, as well as with structural and functional alterations in the thalamus, frontotemporal cortex and subcortical regions.^10-14^ Despite this evidence, however, whether the neurobiological bases underlying these non-motor symptoms in Parkinson’s disease are distinct or may converge on common underlying mechanisms remains poorly elucidated.^7^

To address this question, we conducted a neuroimaging study combining voxel-based morphometry (VBM), coordinate-based network mapping and spatial correlation with neurotransmitter density maps in a large, well-characterized cohort of Parkinson’s disease patients from the Parkinson’s Progression Markers Initiative (PPMI) dataset. By integrating these structural, functional and neurochemical analyses, this study aimed to: (i) identify grey matter (GM) correlates of RBD, depression and anxiety in Parkinson’s disease patients; (ii) characterize the functional networks associated with the atrophy patterns related to each symptom and (iii) determine whether these symptom-specific networks were spatially aligned with the distribution of specific neurotransmitter systems.

## Materials and methods

### Study population

Data used in the preparation of this manuscript were obtained in March 2024 from the PPMI database (www.ppmi-info.org/access-data-specimens/download-data), RRID: SCR_006431. For up-to-date information, visit www.ppmi-info.org. Ethics approval was obtained from the local institutional review boards at each participating site, and written informed consent was provided by all participants.

The study cohort included 668 individuals diagnosed with Parkinson’s disease who had baseline volumetric 3T T1-weighted MRI scans available. Corresponding demographic and clinical data, including age, sex, disease duration, Movement Disorders Society—Unified Parkinson’s Disease Rating Scale (MDS-UPDRS) scores and other relevant variables were extracted from the PPMI database. In addition to standard clinical and demographic variables, clinical scores assessing non-motor symptoms were also considered in Parkinson’s disease patients, including the RBD Screening Questionnaire Score (RBDSQ) for RBD, the Geriatric Depression Scale (GDS) for depressive symptoms and the State-Trait Anxiety Inventory (STAI) for anxiety. Participants with RBD were identified using an RBDSQ Score ≥ 5^15^; those exhibiting depressive symptoms were classified based on a GDS Score ≥ 5^5^; and individuals presenting with anxiety symptoms were identified using an STAI-Trait Score ≥ 39.^6^ However, for imaging analyses, these scores were treated as continuous rather than dichotomous variables. For comparison purposes, demographic and clinical information from a cohort of 188 healthy control (HC) participants was also retrieved from the PPMI database.

### Preprocessing of MRI data

3T MRI scans from Parkinson’s disease patients were spatially normalized to the Diffeomorphic Anatomical Registration Through Exponentiated Lie Algebra template space and segmented into GM, white matter (WM) and CSF using the Computational Anatomy Toolbox (CAT12).^16^ Segmentation was performed using the default CAT12 tissue probability maps and atlas set, which includes the Neuromorphometrics, LONI Probabilistic Brain Atlas 40, Computational Brain Anatomy Laboratory, Thalamus, Thalamic Nuclei and Spatially Unbiased Infratentorial Template templates. The normalized, bias-corrected images were visually inspected to ensure accurate segmentation and alignment. GM maps were subsequently modulated, normalized and smoothed with an 8 mm full width at half-maximum (FWHM) Gaussian kernel, following CAT12 recommendations for VBM analyses (https://neuro-jena.github.io/cat12-help/). To assess the stability of the findings, the analyses were repeated using a 6 mm FWHM smoothing kernel. Sample homogeneity was assessed, and participants exhibiting extreme deviations—defined as a quartic mean *Z*-score exceeding two standard deviations—were identified as statistical outliers and excluded from further analysis (specifically, 30 Parkinson’s disease patients).

### VBM analysis

Three separate multivariate regression VBM analyses were conducted using the SPM12 toolbox^17^ to investigate associations between regional GM volume and clinical scores related to RBD, depression and anxiety in Parkinson’s disease patients. Each clinical score was modelled as a continuous regressor within a multivariate linear regression model. All models included age, sex, education level, disease duration, MDS-UPDRS III, Montreal Cognitive Assessment (MoCA) and total intracranial volume (TIV) as covariates. Additionally, in each analysis, the other two among the three considered non-motor clinical scores were included as covariates to control for their potential confounding effects. Specifically, STAI and GDS scores were considered as covariates in the RBD model; RBDSQ and STAI scores in the depression model; and GDS and RBDSQ scores in the anxiety model. In the RBD model, an additional covariate was included to indicate whether patients were on antidepressant medication at the time of MRI acquisition, as certain antidepressants have been associated with the onset or exacerbation of RBD symptoms.^18^ Statistical inference was performed at the voxel level using a threshold corrected for multiple comparisons with the family-wise error (FWE) method. Results were considered significant at pFWE < 0.05, as no a priori hypotheses were made regarding specific regions of interest. Negative regressions were performed aiming at identifying brain regions where reduced GM density was associated with higher non-motor clinical scores (reflecting more severe symptoms). For each of the three regression models, only clusters exceeding the predefined expected number of voxels per cluster threshold were reported: 31.590 voxels for the RBD model, 34.345 voxels for the anxiety model and 38.123 voxels for the depression model.

### Coordinate-based network mapping

Following the identification of regions where GM volume was significantly associated with the clinical scores through VBM regression analyses, a coordinate-based network mapping approach was performed to characterize the functional brain networks associated with the identified symptom-specific atrophy patterns. This method leverages a connectome derived from resting-state functional 3T-MRI data of a large normative population (*N* = 1000 subjects) to generate connectivity profiles for each seed region identified through VBM analysis. The analysis was performed separately for the three symptoms. For each coordinate emerging from the corresponding symptom-specific VBM regression analyses, spherical regions of interest (ROI) with a 4 mm radius were defined, centred at the corresponding Montreal Neurological Institute (MNI) coordinates^19,20^ and the connectivity network map was generated using the Lead Connectome Mapper toolbox,^21^ based on the functional normative connectome derived from data acquired by the Brain Genomics Superstruct Project (https://dataverse.harvard.edu/dataverse/GSP).^22,23^ This methodology has been previously validated and widely adopted in network mapping approaches,^8,9^ facilitating comparisons across studies. The connectivity map generated from each coordinate was thresholded at a conservative level of *T*  *≥*  *7*, corresponding to an FWE-corrected *P*-value < 10^−6^, in line with previous studies.^19,20,24^ For each considered symptom, the connectivity maps of all significant coordinates from VBM-regression analysis were overlaid to identify brain areas functionally connected to the VBM-derived clusters; regions exhibiting connectivity with at least 70% of the VBM coordinates were identified as part of the functional network underlying that specific symptom. The same procedure was repeated for RBD, depression and anxiety symptoms. In addition, to quantify the spatial similarity between symptom-specific networks, we computed the Dice coefficient for each pair of connectivity maps (RBD–depression, RBD–anxiety and depression–anxiety), providing an index of the degree of overlap ranging from zero (no overlap) to one (perfect overlap). To further characterize the symptom-specific networks identified through coordinate-based network mapping, we assessed their spatial overlap with the canonical large-scale brain networks using a quantitative similarity analysis. Specifically, we compared the thresholded symptom-specific connectivity maps to the seven main functional networks of Yeo atlas^22^ using voxel-wise spatial similarity metrics. Dice coefficients were computed to estimate the degree of overlap between each binarized symptom-specific network and the seven large-scale resting-state networks defined by the Yeo atlas. To determine whether the observed overlap exceeded that expected by chance, statistical significance was assessed using permutation testing based on voxel-wise overlap. For each symptom-related map, random binary masks matched in voxel count were generated within the same analysis space, and overlap with each Yeo network was recomputed to obtain empirical null distributions.^25^ Empirical one-sided *P*-values were then derived from these distributions and corrected for multiple comparisons using the false discovery rate (FDR) (*q* < 0.05). Permutation testing was performed using 10 000 iterations.^26^ The involvement of each canonical network in the spatial overlap with the symptom-related networks was interpreted by jointly considering spatial similarity (Dice), magnitude of enrichment relative to chance and FDR-corrected statistical significance.

### Spatial correlation with neurotransmitter density maps

To investigate the neurochemical substrates underlying the functional networks associated with RBD, depression and anxiety, we evaluated spatial correlations with the normative regional distribution of specific neurotransmitter systems based on PET and SPECT data,^27^ using the JuSpace toolbox (version 1.5). The available neurotransmitter maps covered several key neurochemical systems, including serotonergic [serotonin (5-HT)1a, 5-HT1b, 5-HT2a, 5-HT4, serotonin transporter], endocannabinoid [cannabinoid receptor type 1 (CB1)], cerebral blood flow, dopaminergic [dopamine D1 receptor (D1), D2, dopamine transporter, 6-[18F]fluoro-L-DOPA], GABAergic (gamma-GABA), opioid (kappa opioid receptor, μ-opioid receptor), noradrenergic [noradrenaline transporters (NAT)] and cholinergic (vesicular acetylcholine transporter) glutamatergic (NMDA, metabotropic glutamate receptor subtype 5) systems. These maps were derived from in vivo PET and Single-Photon Emission Computed Tomography (SPECT) imaging of HC cohorts, spatially normalized according to the Neuromorphometrics atlas (http://www.neuromorphometrics.com/), excluding WM and CSF regions. Mean functional connectivity *t*-values within the identified networks for RBD, depression and anxiety were extracted using the Neuromorphometrics atlas for cortical and subcortical regions, and Spearman correlation coefficients were then computed between the z-transformed functional connectivity maps and the neurotransmitter distribution maps provided by the JuSpace toolbox. To assess the statistical significance of the observed spatial correlations, 10 000 permutation tests were conducted using the permutation-based framework implemented in the toolbox, randomizing group labels under the null hypothesis of no association. *P*-values were corrected using the FDR method according to the Benjamini–Hochberg procedure, accounting for the total number of neurotransmitter maps included in the analysis.

### Statistical analysis

Demographic and clinical analyses were performed in Python (version 3.12.7). Group differences between Parkinson’s disease patients and HCs were assessed using independent-samples *t*-tests for continuous variables and χ^2^ tests for categorical variables. Pearson correlation coefficients were computed to assess the associations between RBDSQ, GDS and STAI scores.

For VBM analyses, voxel-wise statistical inference was performed using a general linear model implemented in CAT12, with clinical scores entered as continuous regressors and age, sex, education, disease duration, MDS-UPDRS III, MoCA and TIV included as covariates. Correction for multiple comparisons was applied using the FWE method at *P* < 0.05.

Coordinate-based network mapping was performed using the Lead Connectome Mapper toolbox, and connectivity maps were thresholded at *T* ≥ 7 (corresponding to a voxel-wise FWE-corrected *P* < 10^−6^). Spatial similarity between symptom-related networks and canonical functional Yeo networks was first quantified using Dice coefficients computed in Python (version 3.11), providing a descriptive measure of spatial overlap. To assess whether the observed overlap exceeded that expected by chance, statistical significance was evaluated using permutation testing based on voxel-wise overlap. Specifically, for each symptom-related map, random binary masks matched in voxel count were generated within the common analysis space, and their overlap with each Yeo network was recomputed to obtain empirical null distributions using 10 000 permutations. Empirical one-sided *P*-values were derived from these null distributions and corrected for multiple comparisons using the FDR (*q* < 0.05).

Spatial correlations between symptom-specific functional networks and neurotransmitter distribution maps were assessed using JuSpace (version 1.5). Spearman correlation coefficients were computed between z-transformed connectivity values and normative PET/SPECT neurotransmitter maps. Statistical significance was tested using 10 000 permutations, and *P*-values were corrected for multiple comparisons using the FDR according to the Benjamini–Hochberg procedure.

### Image acquisition and processing tools

**Figure 1:**
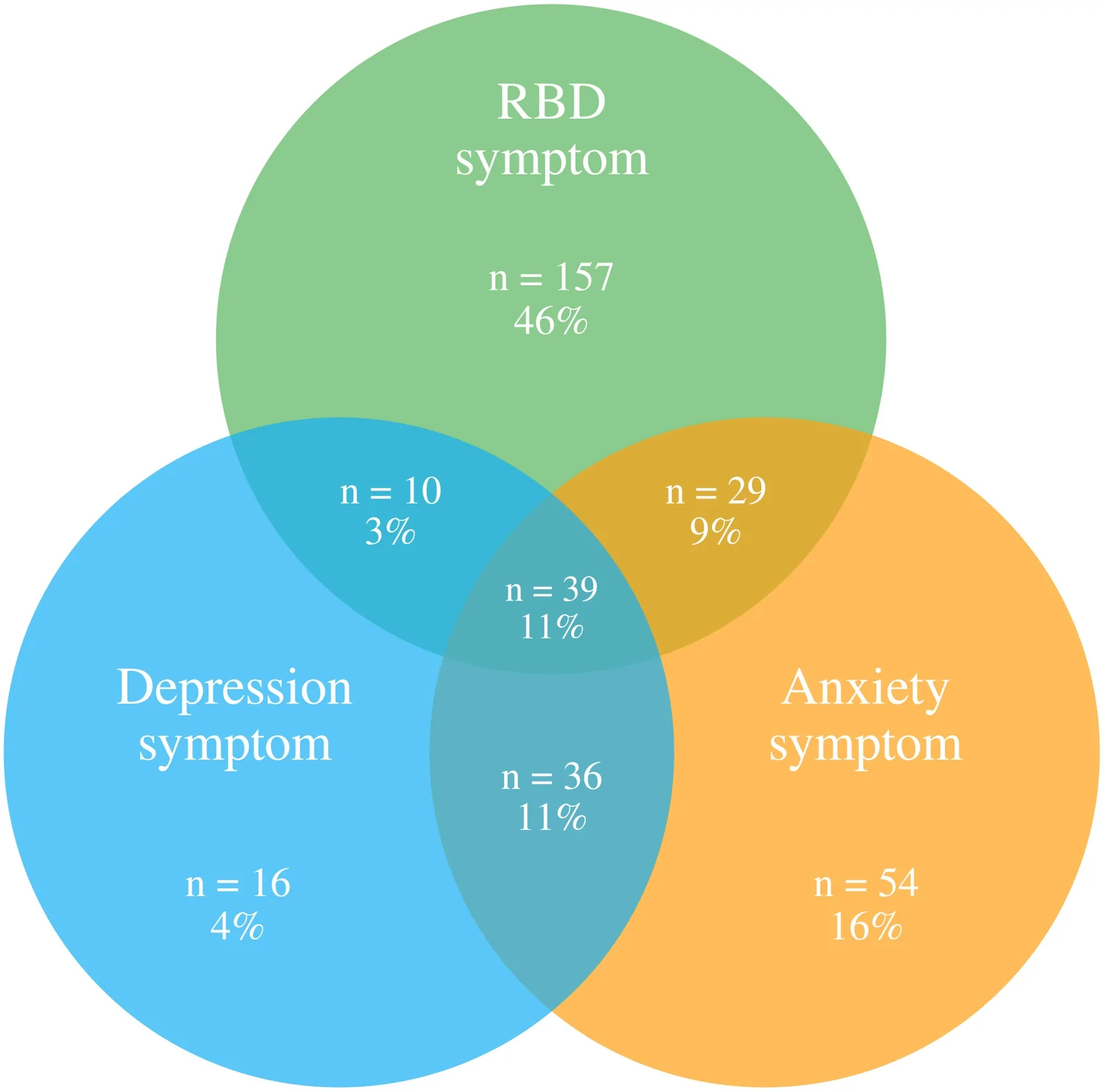
**Venn diagram showing the distribution and overlap of non-motor symptoms in the Parkinson’s disease cohort.** The diagram illustrates the number of patients presenting with symptoms suggestive of REM sleep behaviour disorder (RBDSQ Score ≥ 5), depressive symptoms (GDS Score ≥ 5) and anxiety symptoms (STAI-Trait Score ≥ 39), as described in the Materials and methods. The intersections represent the number of patients experiencing symptoms belonging to multiple non-motor domains. RBDSQ = REM Sleep Behaviour Disorder Screening Questionnaire; STAI = State-Trait Anxiety Inventory; GDS = Geriatric Depression Scale.

**Figure 2:**
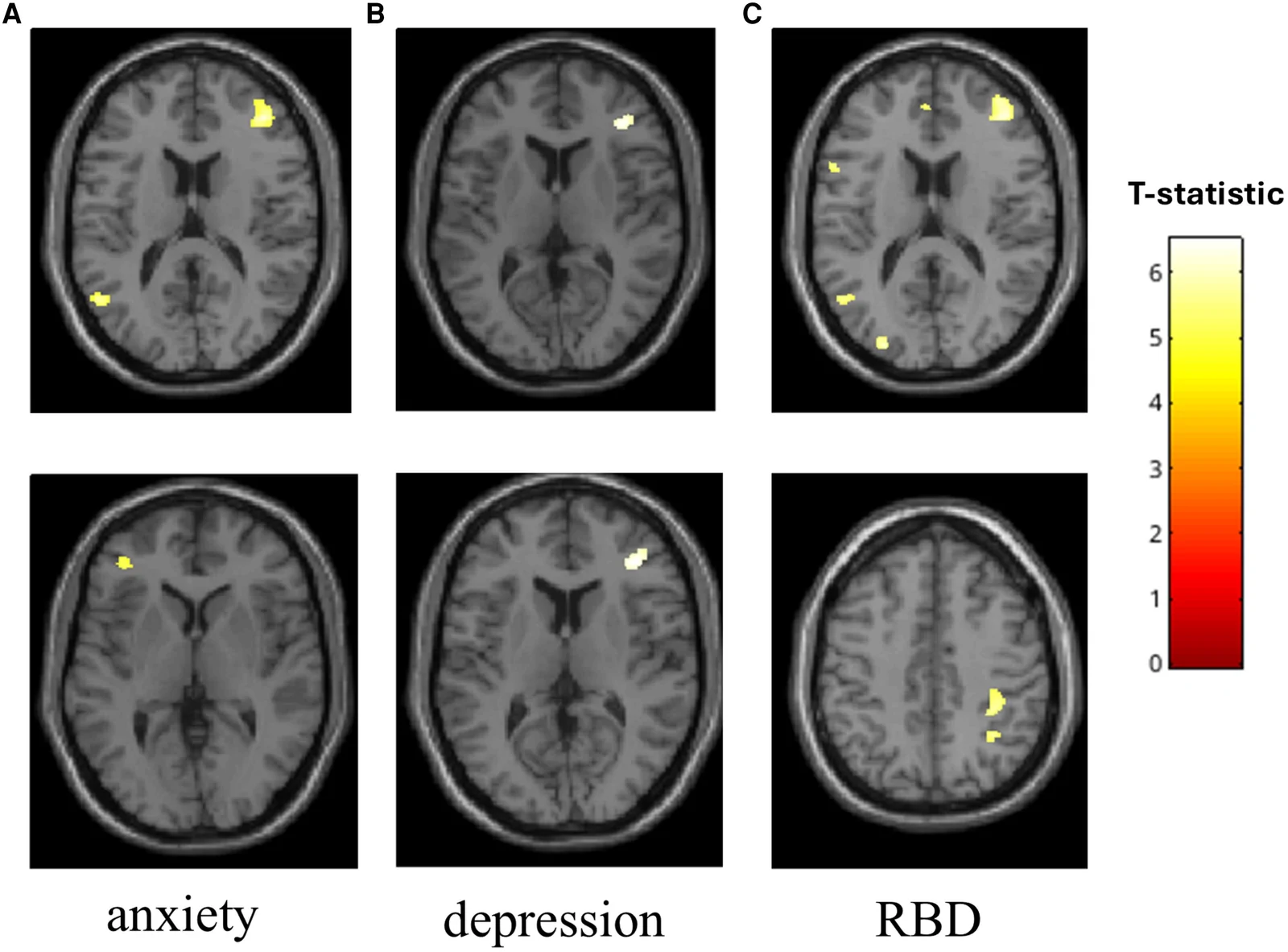
**Representative axial slices showing results from Voxel Based Morphometry regression analyses.** (**A**) Regions showing negative associations with STAI scores (anxiety). (**B**) Regions negatively associated with GDS scores (depression). (**C**) Regions negatively associated with RBDSQ scores (RBD). The scale bar indicates t-values, with higher absolute t-values reflecting stronger negative associations. Images are displayed in neurological orientation (left = left, right = right; anterior = top, posterior = bottom). All results are thresholded at *P* < 0.05, corrected for multiple comparisons using family-wise error (FWE) correction at the voxel level. Voxel-based morphometry regression analyses were performed in *N* = 638 Parkinson’s disease patients. Experimental unit: individual patient MRI scan. RBDSQ = REM Sleep Behaviour Disorder Screening Questionnaire; STAI = State-Trait Anxiety Inventory; GDS = Geriatric Depression Scale.

**Figure 3:**
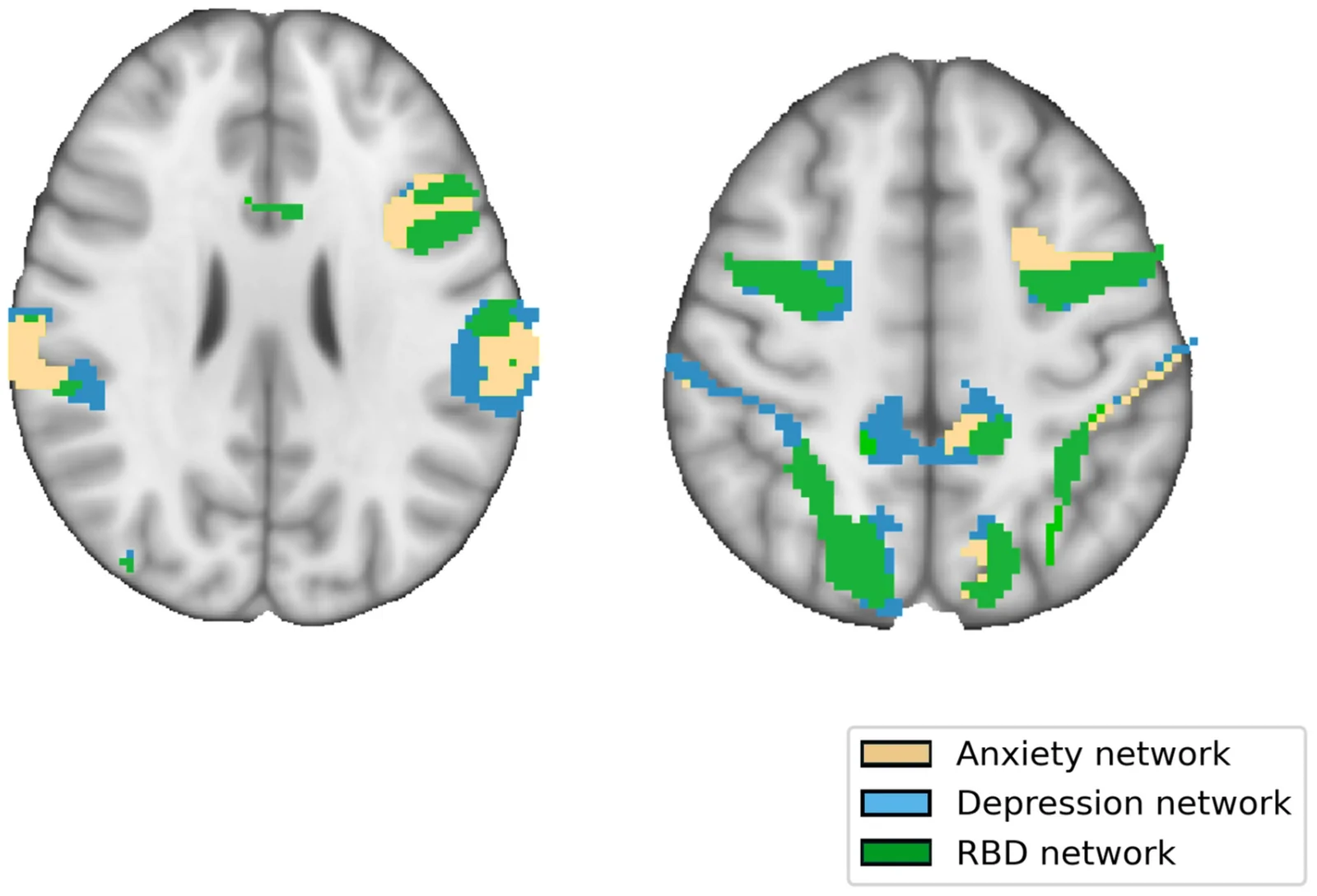
**Representative images showing the largest overlap regions resulting from the overlay of coordinate-based network maps for RBD, depression and anxiety in patients with Parkinson’s disease.** Binarized connectivity maps derived from voxel-based morphometry (VBM) coordinates associated with each of the three considered non-motor symptoms (REM sleep behaviour disorder (RBD), depression and anxiety) revealed substantial overlap across cortical regions, including temporal, insular, cingulate and parietal areas. Connectivity maps were generated using seed-based functional connectivity *T* maps from a normative resting-state connectome. Each connectivity map was thresholded at *T* ≥ 7, corresponding to a family-wise error-corrected significance level of *P* < 10^−6^. Colours indicate network maps associated with RBD (green), depression (light blue) and anxiety (orange). The whole overlay and individual maps are shown in Supplementary materials. Images are displayed in neurological orientation (left = left, right = right; anterior = top, posterior = bottom). Functional networks were mapped using VBM-derived coordinates from *N* = 638 Parkinson’s disease patients, projected onto a normative connectome (*N* = 1000 healthy controls). Experimental unit: MNI coordinate.

**Figure 4:**
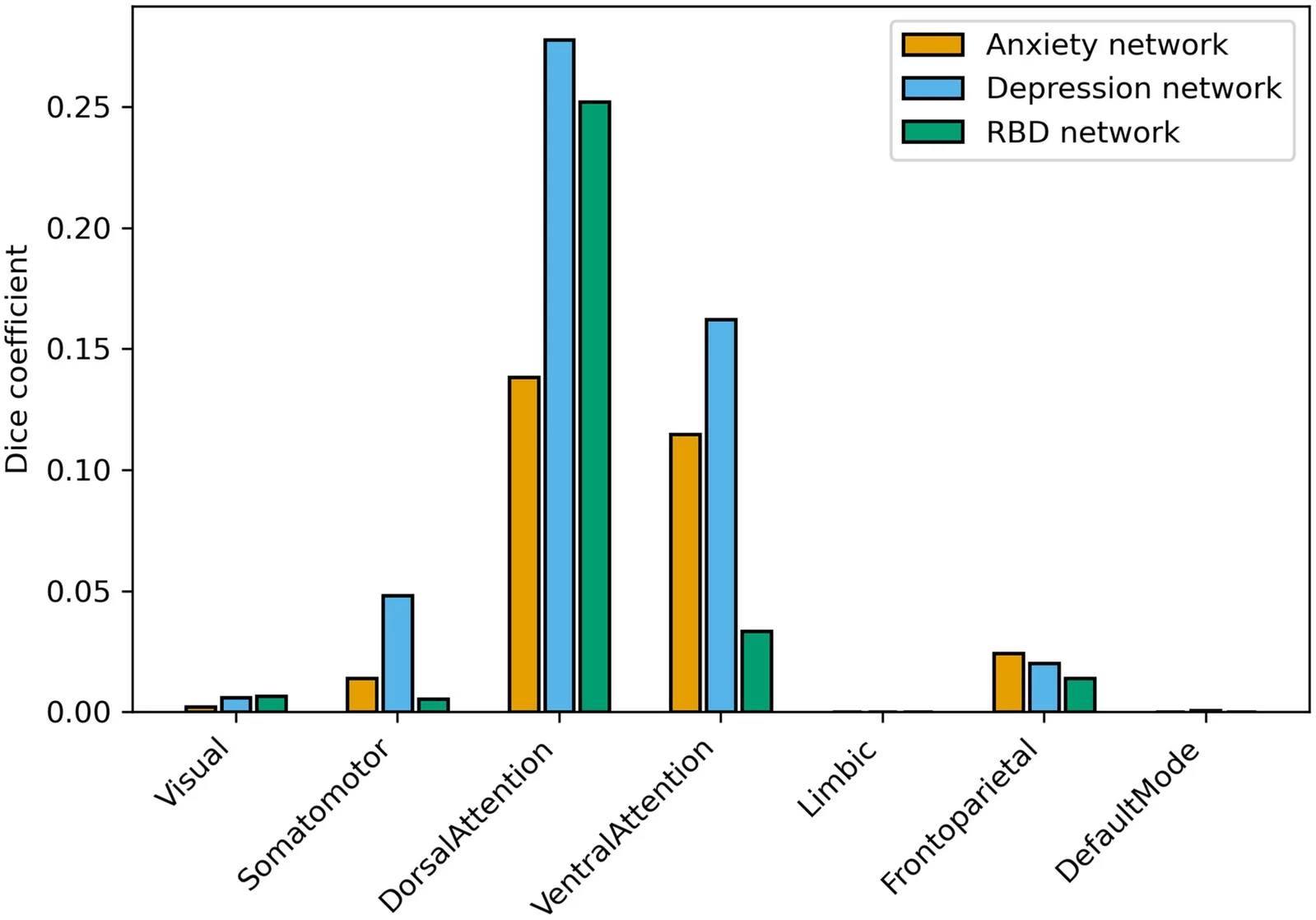
**Bar plot showing the spatial overlap between functional connectivity networks associated with REM sleep behaviour disorder (RBD) (green), depression (light blue) and anxiety (light orange) in Parkinson’s disease patients and canonical large-scale brain networks defined by the Yeo atlas.** The *y*-axis displays the Dice coefficient, a measure of spatial similarity ranging from 0 (no overlap) to 1 (complete overlap). The *x*-axis lists the seven large-scale functional networks. Values represent voxel-wise spatial overlap between the binarized symptom-specific networks and each Yeo network. Dice values are intended as a comparative index of relative affinity across networks rather than as a measure of absolute correspondence. Functional connectivity networks associated with RBD, depression and anxiety were derived from *N* = 638 Parkinson’s disease patients. Experimental unit: voxel.

**Figure 5:**
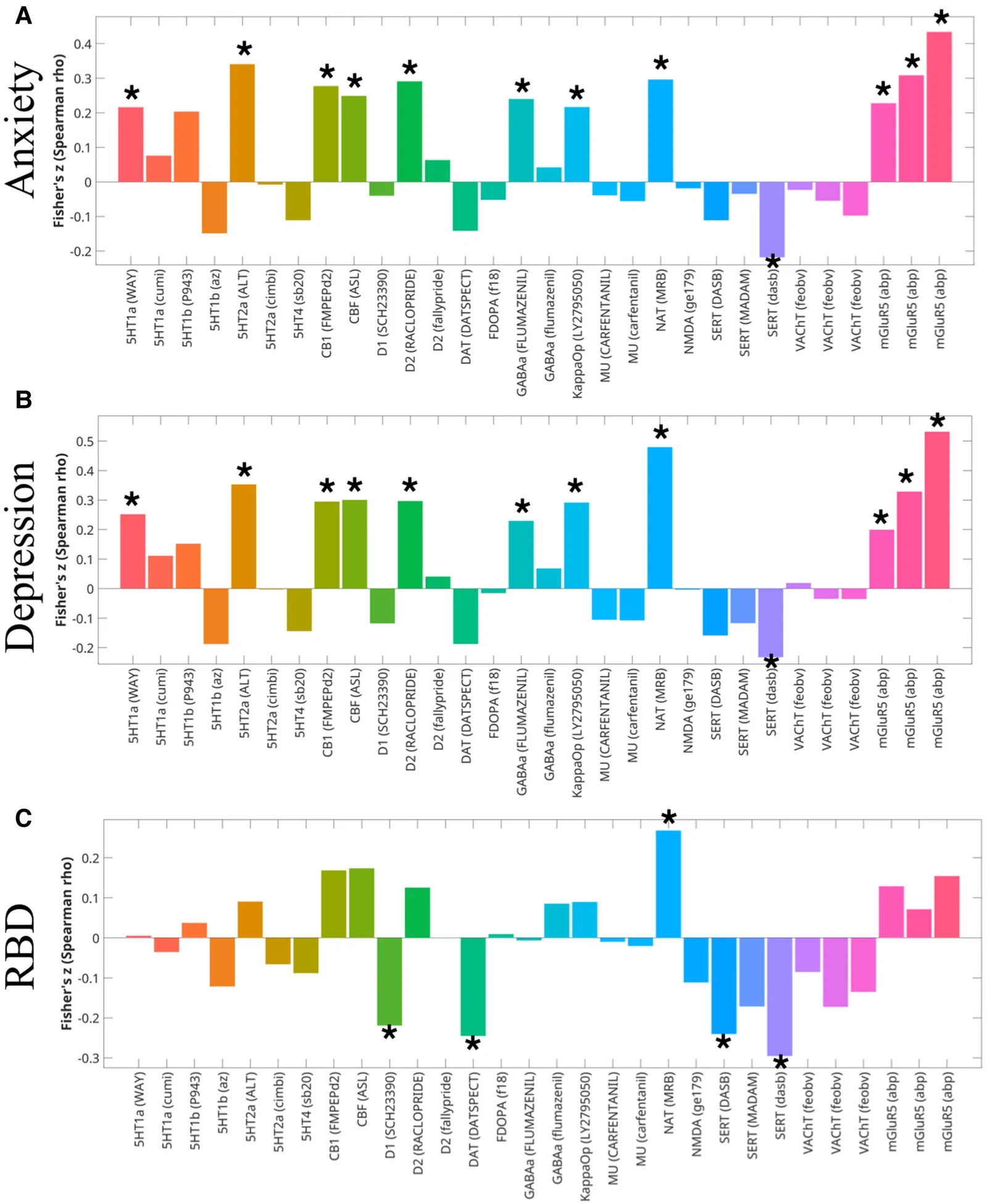
**Spatial correlation analysis between functional connectivity networks and normative density maps of several neurotransmitter systems in Parkinson’s disease patients.** (**A**) Functional connectivity network associated with anxiety. (**B**) Functional connectivity network associated with depression. (**C**) Functional connectivity network associated with REM sleep behaviour disorder (RBD). Neurotransmitter systems included serotonergic (5-HT1a, 5-HT1b, 5-HT2a, 5-HT4, SERT), endocannabinoid (CB1), cerebral blood flow (CBF), dopaminergic (D1, D2, DAT, FDOPA), GABAergic (GABAa), opioid (KappaOp, MU), noradrenergic (NAT), cholinergic (VAChT), glutamatergic (NMDA, mGluR5) systems. Bar plots show Spearman correlation between functional connectivity values and neurotransmitter density maps, using z-transformed coefficients. Statistical significance was assessed using 10 000 permutations with false discovery rate (FDR) correction across neurotransmitter maps (pFDR < 0.05); asterisks indicated statistically significant correlations. Spatial correlations were computed between symptom-specific functional networks (derived from *N* = 638 Parkinson’s disease patients) and normative neurotransmitter density maps. Experimental unit: brain region (Neuromorphometrics atlas, excluding white matter and CSF regions). RBDSQ = REM Sleep Behaviour Disorder Screening Questionnaire; STAI = State-Trait Anxiety Inventory; GDS = Geriatric Depression Scale; 5-HT = serotonin; CB1 = cannabinoid receptor type 1; CBF = cerebral blood flow; D1 = dopamine D1 receptor; D2 = dopamine D2 receptor; DAT = dopamine transporter; FDOPA = 6-[18F]fluoro-L-DOPA; GABAa = gamma-GABA; KappaOp = kappa opioid receptor; MU = μ-opioid receptor; NAT = norepinephrine transporter; SERT = serotonin transporter; VAChT = vesicular acetylcholine transporter; mGluR5 = metabotropic glutamate receptor subtype 5. **Alt text**:

All figures were generated in accordance with current community standards to ensure accurate representation of the original data. Figure 1 was created with Canva, Fig. 2 with CAT12 toolbox, Fig. 3 with FMRIB Software Library eyes (FSLeyes), Fig. 4 with Python (version 3.11.8) and Fig. 5 with the JuSpace toolbox. All Supplementary figures were prepared with FSLeyes. All brain images were displayed on the MNI152 T1 brain template (2 mm resolution) as distributed in FSL.^28^ Collages and schematic layouts were designed in OmniGraffle, ensuring that the scientific content of the images was preserved.

## Results

The final Parkinson’s disease population included 638 patients with available MRI and clinical scores for the symptoms of interest from the PPMI database. Parkinson’s disease patients showed significantly higher scores than HC on the RBDSQ, GDS and STAI scales (all *P*  *<*  *0.001*), indicating a greater burden of non-motor symptoms, despite similar age and sex distribution (Table 1). Most Parkinson’s disease patients (341/638 = 53.4%) showed at least one of the considered non-motor symptoms. Among these, 33.4% had more than one symptom, and 11.4% had all three considered non-motor symptoms (Fig. 1). Consistent with these findings, correlation analyses confirmed significant associations among all three non-motor symptom domains (Table 2).

**Table 1:** Baseline demographic and clinical data of Parkinson’s disease patients and healthy controls

Data	Parkinson’s disease (*N* = 638)	HC (*N* = 188)	*P*-value
**Sex, (M/F)**	246/392	68/120	0.192^a^
**Age at examination, years^b^**	62.9 ± 9.6	61.5 ± 11.6	0.262^c^
**Education, years^b^**	15.7 ± 2.9	16.3 ± 2.6	0.049^c^
**Disease duration, years^b^**	0.9 ± 1.1	**-**	**-**
**MDS-UPDRS-I^b^**	6.2 ± 4.6	**-**	**-**
**MDS-UPDRS-II^b^**	6.3 ± 4.6	**-**	**-**
**MDS-UPDRS-III^b^**	22.8 ± 10.0	**-**	**-**
**RBDSQ^b^**	4.1 ± 2.9	2.7 ± 2.4	**<0.001** ^c^
**GDS^b^**	2.4 ± 2.6	1.2 ± 2.0	**<0.001** ^c^
**STAI^b^**	64.7 ± 18.6	55.5 ± 13.0	**<0.001** ^c^
**MoCA^b^**	26.8 ± 2.5	28.5 ± 1.5	**<0.001** ^c^

Bold values indicate highly statistically significant results (*P* < 0.001).

HC = healthy controls; MDS-UPDRS = Movement Disorders Society—Unified Parkinson’s Disease Rating Scale—part I, II, III; RBDSQ = REM Sleep Behaviour Disorder Screening Questionnaire; GDS = Geriatric Depression Scale; STAI = State-Trait Anxiety Inventory; MoCA = Montreal Cognitive Assessment; M = male; F = female.

^a^Fisher’s exact test.

^b^Data are expressed as mean ± standard deviation.

^c^ *t*-test or Wilcoxon rank sum test.

**Table 2:** Correlations across non-motor symptom measures in Parkinson’s disease patients

Pairwise correlation	Pearson’s *r*	*P*-value
**RBDSQ—STAI**	0.198	**<0.001**
**RBDSQ—GDS**	0.187	**<0.001**
**STAI—GDS**	0.671	**<0.001**

Bold values indicate highly statistically significant results (*P* < 0.001).

RBDSQ = REM Sleep Behaviour Disorder Screening Questionnaire; STAI = State-Trait Anxiety Inventory; GDS = Geriatric Depression Scale.

### VBM regression analyses

VBM analyses revealed significant negative associations between regional GM volumes and clinical scores (RBDSQ, GDS and STAI) using an FWE-corrected threshold of *P*  *<*  *0.05.* Higher RBDSQ scores were associated with reduced GM volumes in several cortical regions, including the right middle frontal gyrus, the bilateral middle temporal gyri, right inferior parietal gyrus, left anterior cingulate cortex, left inferior frontal gyrus and left middle occipital gyrus, with the largest cluster located in the right middle frontal gyrus (Supplementary Table 1). GDS and STAI scores showed negative correlations mainly with GM volume in the left middle temporal gyrus and right middle frontal gyrus. These two regions were also identified in the RBD VBM regression analysis as stated above, emerging as key regions across all three symptom domains. Full details of the significant clusters and corresponding MNI coordinates are provided in Supplementary Table 1. Representative images illustrating the VBM regression results are shown in Fig. 2. Comparable results were obtained when analyses were repeated using a 6 mm smoothing kernel, with identical peak coordinates.

### Coordinate-based network mapping results

To delineate the functional connectivity profiles associated with the structural alterations identified through VBM analyses, we performed a coordinate-based network mapping approach separately for RBD, depression and anxiety. For each symptom domain, the connectivity maps from each VBM coordinate were binarized by applying a threshold of *T* ≥ 7 consistent across all analyses and subsequently overlaid to identify brain areas functionally connected to most of the symptom-specific VBM-derived clusters. For the RBD analysis, the final symptom-specific connectivity network map resulted from the overlap across 70% of the maps derived from single VBM clusters. For STAI and GDS analyses, a lower number of VBM coordinates was previously identified (five and two clusters, respectively), thus the final network map was defined as the overlap across all (100%) single VBM cluster connectivity maps. Despite slight symptom-specific variations, the resulting network maps showed a large overlap across the three symptoms in several cortical regions, including the bilateral inferior and middle temporal gyri, bilateral insular cortices, bilateral supramarginal gyri, bilateral precuneus cortex, bilateral postcentral gyrus, left precentral and middle frontal gyrus, bilateral cingulate gyrus and bilateral angular gyrus, suggesting that these symptoms may share a common underlying functional architecture (Supplementary Fig. 1). The whole network maps are shown in Supplementary Figs 1–4. Representative images focusing on the largest overlapping regions are provided in Fig. 3. To quantify the spatial similarity between symptom domains, we computed the Dice coefficient for each pair of networks. The highest similarity was observed between the anxiety and depression networks (Dice = 0.49) and the depression and RBD networks (Dice = 0.47), followed by the anxiety and RBD networks (Dice = 0.29), overall reflecting the observed correlations among clinical symptoms. We then investigated whether the identified symptom-specific networks preferentially overlapped with canonical large-scale functional systems defined by the Yeo atlas. Among the seven canonical Yeo networks, the Dorsal Attention network (DAN) showed the greatest spatial correspondence with all three symptom-related networks, as indicated by the highest Dice coefficients (Fig. 4). Anxiety- and depression-related networks also showed relatively high affinity with the Ventral Attention network (VAN).^29^ To formally assess network specificity, statistical significance was evaluated using permutation testing on voxel-wise overlap with 10 000 random binary masks matched in voxel count, followed by FDR correction for multiple comparisons. In addition to Dice coefficients, we quantified the magnitude of overlap relative to chance using fold enrichment. These results are illustrated in Supplementary Fig. 5. Importantly, the observed overlap pattern was not uniformly distributed across canonical networks but showed significant enrichment within attention-related systems. The involvement of each canonical network in the spatial overlap with the symptom-related networks was interpreted by jointly considering spatial similarity (Dice coefficient), magnitude of enrichment relative to chance and FDR-corrected statistical significance. Within this framework, the DAN consistently exhibited the strongest and most consistent enrichment across all three symptom-related networks, while the VAN showed significant enrichment for the anxiety- and depression-related networks. In contrast, networks showing low Dice coefficients were not considered to reflect meaningful preferential spatial correspondence, even when statistical significance was observed. The degree of spatial similarity, as reflected by the Dice coefficient, is shown in Fig. 4, while the similar topographical distribution across these networks is presented in Supplementary Fig. 6.

### Spatial correlation with neurotransmitter density maps

Finally, we carried out a spatial correlation analysis between the three symptom-specific functional connectivity network maps and the regional distribution of several neurotransmitter systems based on PET and SPECT normative data, and we identified patterns of significant positive spatial correlations (*z* > 0.2, pFDR < 0.05). The RBD-associated network (Fig. 5A) showed a significant spatial correlation with the NAT density map, suggesting a predominant involvement of the noradrenergic system. The networks associated with depression (Fig. 5B) and anxiety (Fig. 5C) in Parkinson’s disease showed significant positive correlations with a broader range of neurotransmitter systems, including serotonergic, dopaminergic, endocannabinoid, glutamatergic and GABAergic systems and the noradrenergic (NAT) system. This latter NAT neurotransmitter map was thus associated with all the RBD-, depression- and anxiety-associated networks, suggesting a shared role of the noradrenergic system in all these symptoms.

## Discussion

The present work provides a novel multistep imaging approach integrating VBM, coordinate-based network mapping and spatial correlation with neurotransmitter density maps to identify the neurobiological bases of specific symptoms or diseases. In this study, we employed this neuroimaging pipeline to investigate the anatomical and functional substrates of RBD, depression and anxiety in a large cohort of early-stage Parkinson’s disease patients from the PPMI, and we found that these non-motor symptoms were linked to largely overlapping functional connectivity networks involving attention networks and showing consistent spatial association with the noradrenergic system, in line with the frequent co-occurrence of these symptoms in Parkinson’s disease.

Over the past decade, a growing body of research has reshaped our understanding of Parkinson’s disease by emphasizing the central role of non-motor symptoms in its clinical and pathophysiological profile.^2,3^ Increasing attention has been directed towards symptoms such as RBD, depression and anxiety, which are commonly observed in the early stage of the disease, highly prevalent and often co-occurring in Parkinson’s disease patients, as observed in the large international Parkinson’s disease cohort analysed in the current work. In this study, we tested the hypothesis that these symptoms may have common underlying neurobiological bases, reflecting their clinical association.^7,30^ We first employed VBM regression to identify associations between regional GM volumes and clinical scores (RBDSQ, GDS and STAI), and we identified distinct patterns of VBM clusters converging in the left middle temporal gyrus and right middle frontal gyrus—regions that were previously identified in depressed Parkinson’s disease patients^31^ and that emerged here as shared anatomical substrates across all three non-motor symptom domains. In addition to these two clusters, higher burden of RBD symptoms was associated with volumetric reductions also in the right inferior parietal gyrus, left anterior cingulate cortex and left inferior frontal gyrus—findings consistent with previous studies reporting structural changes in similar regions in Parkinson’s disease patients with RBD.^32^ Although these symptoms have been investigated separately in previous morphometric studies, each has been linked to GM reductions in frontotemporal and cingulate regions.^9,33,34^ These observations collectively support the idea that several non-motor symptoms in Parkinson’s disease may arise from shared structural vulnerabilities within associative cortical systems.

To investigate whether these morphological changes reflected coherent functional convergence and to which functional networks these GM atrophy patterns were related, we employed a coordinate-based network mapping approach using a large normative resting-state connectome. This method allowed us to identify the brain regions functionally connected to the symptom-specific VBM clusters of GM alterations and revealed that the regions identified through VBM analyses for RBD, depression and anxiety were mapped onto widely overlapping brain networks encompassing the inferior temporal gyri, insular cortex, supramarginal and angular gyri, precuneus, cingulate cortex and middle frontal areas. Some slight disease-specific differences across connectivity networks were observed. For example, the depression-related network extended to the temporal pole and orbitofrontal cortex, consistent with the involvement of prefrontal–limbic circuits described in prior imaging studies of mood symptoms in Parkinson’s disease.^35,36^ For anxiety, the mapped network included the parietal operculum and anterior cingulate cortex—structures commonly implicated in the fear circuit and the cortico–striato–thalamo–cortical loop known to be disrupted in anxious Parkinson’s disease patients.^6^ Finally, the network associated with RBD extended to the bilateral thalamus, paracingulate gyrus and motor cortical areas, in line with previous studies reporting thalamic degeneration and frontoparietal network alterations in RBD.^37,38^ Despite these slight differences, however, the three networks showed significant topological overlap among each other and, among canonical large-scale brain systems, exhibited the greatest spatial affinity with the DAN (for all three symptoms) and the VAN (for anxiety and depression). While the VAN is primarily involved in stimulus-driven attention, salience detection and reorienting to behaviourally relevant or unexpected stimuli,^29,39^ the DAN supports goal-directed attentional control and the regulation of externally oriented behaviour,^39-41^ potentially contributing to difficulties in shifting attention, heightened emotional reactivity and poor regulation of arousal in Parkinson’s disease. Overall, these findings demonstrate connectivity network overlap, which may partly account for the clinical association among RBD, depression and anxiety in Parkinson’s disease. The preferential spatial affinity of symptom-related networks onto VAN and DAN suggests that interventions capable of modulating these attentional systems might offer benefits for several non-motor symptoms. Among promising non-invasive strategies, transcranial direct current stimulation (tDCS) has shown encouraging results. Notably, anodal tDCS applied to the prefrontal cortex in Parkinson’s disease reduced mood and anxiety symptoms, enhanced attentional control and importantly, improved sleep scores in early-onset Parkinson’s disease patients—an outcome that may indirectly reflect amelioration of RBD-related sleep disturbances.^42^ Additionally, motor cortex tDCS has demonstrated improvements in non-motor symptoms overall, possibly through its effects on cortico–subcortical circuits involved in both sleep and mood regulation.^43^ The present findings should be interpreted within a multifactorial framework. The structural and functional alterations identified here likely reflect a shared neurobiological vulnerability across RBD, depression and anxiety, rather than deterministic mechanisms underlying their clinical manifestation. The observation that only a few patients express all three symptoms simultaneously suggests that additional modulatory factors—including genetic predisposition, environmental exposures, personality traits, sleep quality and psychosocial context—interact with this shared neural substrate to shape the individual clinical phenotype. Future neurophysiopathological studies integrating longitudinal imaging, genetic profiling and sleep–wake assessments will be essential to disentangle the relative contribution of imaging vulnerability and modulatory factors in shaping the individual risk for non-motor symptoms.

Finally, this study found evidence that the anatomical and functional convergence observed across symptom domains translated into a consistent pattern of neurotransmitter involvement. Although anxiety and depression-associated networks showed associations with the distribution of several neurotransmitters, in line with the existing literature,^41,44^ one of the strongest positive spatial correlations involving all three symptom-specific networks was with the NAT density map. This result aligns with growing evidence implicating noradrenergic dysfunction, particularly within the locus coeruleus and its cortical projections, in a wide range of non-motor symptoms in Parkinson’s disease.^45^ Previous PET imaging studies in idiopathic RBD have demonstrated reduced NAT availability in brainstem and cortical regions, indicating that the loss of noradrenergic input may play a crucial role in the pathogenesis of RBD-related behaviours.^46^ Similarly, reduced noradrenaline levels have been consistently associated with depressive and anxious phenotypes, both in idiopathic mood disorders and in Parkinson’s disease.^47^ Beyond regional NAT reductions, recent neurobiological models suggest that the noradrenergic system plays a key modulatory role across several large-scale circuits involved in arousal, emotional regulation and REM sleep control. Noradrenaline dynamically adjusts cortical excitability, stress responsivity and attentional gain, and its loss leads to impaired top–down regulation of limbic responses, heightened anxiety, reduced mood resilience and abnormalities in REM atonia mechanisms.^48-50^ These mechanistic insights provide a coherent explanation for why noradrenergic degeneration can simultaneously influence RBD-related behaviours, mood disturbances and anxiety symptoms, offering a unifying neurochemical framework that aligns with the converging network alterations identified in our analyses.

Several methodological strengths support the robustness of our findings. First, our study benefits from a large international sample size of Parkinson’s disease patients and high-resolution structural imaging data obtained from the PPMI dataset. Second, Parkinson’s disease patients were in the early disease stage (minimizing the impact of disease progression on brain atrophy), and all analyses were rigorously controlled for a comprehensive set of potential confounders, including age, sex, education, disease duration, UPDRS-III and MoCA, thereby minimizing confounding effects related to other aspects of the disease. In addition, we explored and removed the possible effect of antidepressant medications on RBD, often overlooked in previous studies. Third, we used rigorous statistical correction procedures, with all VBM results surviving FWE correction and connectivity maps surviving at a conservative threshold of *T* ≥ 7 (corresponding to FWE-corrected *P*-value <10^−6^), as recommended in previous studies.^20,24,51^ Fourth, the integration of structural imaging findings with normative functional connectivity data allowed us to explore the broader functional context of regional GM changes; moreover, by correlating symptom-related network topographies with normative maps of neurotransmitter distribution, we were able to link large-scale network alterations to specific neurochemical systems, thereby adding a molecular dimension to the interpretation of our functional and anatomical findings. Importantly, the statistical significance of these spatial correlations was assessed using permutation testing with 10 000 iterations, and results were corrected for multiple comparisons using the FDR according to the Benjamini–Hochberg procedure, further ensuring the robustness of the observed associations.

Notably, beyond the specific findings related to RBD, depression and anxiety in Parkinson’s disease, this study introduces a conceptual and methodological framework integrating well-established and validated techniques including VBM, coordinate-based connectivity network mapping and spatial correlation with neurotransmitter density maps, which requires only T1-weighted MRI data and may be suitable for broader use to investigate the anatomical, functional and neurochemical correlates of neurological symptoms or disorders. Nonetheless, several limitations must be acknowledged. From the methodological perspective, as in previous studies adopting coordinate-based network mapping,^24,52^ a major limitation is the reliance on a normative connectome derived from healthy people, which may not fully capture disease-specific reorganization of functional networks in Parkinson’s disease. While this approach allows for a standardized and reproducible assessment of functional architecture, it precludes the direct investigation of individual variability or dynamic changes in connectivity related to disease progression. Second, it should be acknowledged that VBM signal differences reflect local variations in GM concentration and may be influenced not only by true atrophy but also by methodological factors such as cortical folding, registration accuracy and partial volume effects. For this reason, VBM findings should be interpreted as indicating regional structural differences rather than direct evidence of neuronal loss. Third, the use of questionnaire-based assessments, although validated, may raise concerns on clinical measurement reliability—particularly for symptoms such as RBD, which ideally require polysomnographic confirmation. To avoid possible errors in patient group allocation based on questionnaire data, we decided to employ VBM regressions to identify associations between imaging data and clinical scores rather than stratifying patients into different subgroups. Fourth, it should be noted that the neuroimaging findings identified here explain only a portion of the variance in symptom expression; the individual clinical phenotype is likely shaped by the interaction between these structural and functional vulnerabilities and non-imaging factors such as genetic predisposition, environmental exposures and psychosocial conditions. Another important limitation concerns the interpretation of spatial correlations with neurotransmitter maps. While permutation-based statistics were applied to control for multiple comparisons, the neurotransmitter maps included in the analysis are not independent and may exhibit substantial spatial collinearity. Therefore, the observed associations should not be interpreted as reflecting selective or exclusive involvement of specific neurotransmitter systems. Future studies using multivariate approaches will be necessary to clarify the specificity of these neurochemical associations. Finally, although the JuSpace toolbox provides an extensive and heterogeneous set of PET/SPECT maps, it does not cover the full range of neurotransmitter systems present in the human brain. In the case of dopamine, the available maps are mainly based on tracers binding to striatal dopamine receptors and transporters, predominantly reflecting motor and mesolimbic pathways rather than the broader spectrum of dopaminergic projections.

In conclusion, our findings demonstrated that RBD, depression and anxiety in early-stage Parkinson’s disease patients were associated with converging patterns of GM atrophy, which localized within a shared functional network and exhibited strong spatial alignment with the NAT noradrenergic system. We developed a novel framework integrating structural, functional and neurochemical data, which allowed us to provide a more comprehensive view of the neurobiological mechanisms underlying these non-motor symptoms in Parkinson’s disease and may be of interest for future research.

## Supplementary Material

fcag316_Supplementary_Data

## Data Availability

The data supporting the findings of this study are openly available in the Image and Data Archive at https://ida.loni.usc.edu/login.jsp?project=PPMI. All custom scripts used for data analysis in this study are available on GitHub at the following repository: https://github.com/chiaracamastra/Coordinate-based-network-mapping. Additional analyses were performed using established MATLAB-based toolboxes (SPM12, CAT12, Lead Connectome Mapper, JuSpace), freely available and downloadable from their respective repositories and installed within the MATLAB environment.
