# Supplementary material for: Structural, functional and neurochemical imaging mapping of non-motor symptoms in Parkinson’s disease
Source: Brain Commun. 2026 Aug 18;8(4):fcag316. doi: 10.1093/braincomms/fcag316 (PMC13504651; doi:10.1093/braincomms/fcag316)
Supplement: Supplementary file 1 [file fcag316_supplementary_data.docx]

**Supplementary Table 1 Brain regions showing significant negative correlations between grey matter volumes and clinical measures (RBDSQ, GDS, STAI) based on voxel-based morphometry.**

| **Negative VBM regression** | **MNI coordinate** | **N. of voxels** | **Side** | **Lobe** | **Region** |
| --- | --- | --- | --- | --- | --- |
| RBDSQ | 42, 42, 15 | 624 | Right | Frontal | Middel Frontal Gyrus |
|  | -54, 12, 18 | 115 | Left | Frontal | Inferior Frontal Gyrus |
|  | -45, 4, 21 | 115 | Left | Frontal | Inferior Frontal Gyrus |
|  | -27, -87, 15 | 147 | Left | Occipital | Middel Occipital Gyrus |
|  | 30, -40, 42 | 277 | Right | - | Cerebral White Matter |
|  | -44, -36, 2 | 52 | Left | - |  |
|  | 34, -52, 45 | 68 | Right | Parietal | Inferior Parietal Gyrus |
|  | -50, -63, 16 | 53 | Left | Temporal | Middle Temporal Gyrus |
|  | -2, 46, 15 | 51 | Left | Limbic | Anterior Cingulate Cortex |
|  | 0, 48, 6 | 51 | Left | Limbic | Anterior Cingulate Cortex |
| GDS | -51, -52, 15 | 66 | Left | Temporal | Middle Temporal Gyrus |
|  | 40, 40, 14 | 168 | Right | Frontal | Middle Frontal Gyrus |
| STAI | -51, -62, 15 | 121 | Left | Temporal | Middle Temporal Gyrus |
|  | 42, 42, 15 | 671 | Right | Frontal | Middle Frontal Gyrus |
|  | 28, -40, 40 | 102 | Right | - | Cerebral White Matter |
|  | 36, -36, 36 | 102 | Right | - |  |
|  | -38, 45, 0 | 83 | Left | - | Cerebral White Matter |

Abbreviations: MNI = Montreal Neurological Institute; RBDSQ = REM Sleep Behavior Disorder Screening Questionnaire; GDS = Geriatric Depression Scale; STAI = State-Trait Anxiety Inventory.

All differences were corrected at p_FWE_ < 0.05. Age, sex, disease duration, education level, UPDRS-III,MoCA, and TIV were included as covariates in all VBM analyses. Additionally, in each analysis, the other two clinical scores were included as covariates to control for their potential confounding effects.

**Supplementary Figure 1. Overlay of the coordinate-based connectivity network maps associated with each of the three considered non-motor symptoms (REM Sleep Behaviour Disorder (RBD), depression and anxiety) in patients with Parkinson’s disease.** Connectivity maps were generated using seed-based functional connectivity T-maps derived from a large normative resting-state fMRI connectome (n=1000 healthy controls). For each Voxel-Based Morphometry-derived coordinate, the corresponding T-map was thresholded at T ≥ 7, equivalent to a family-wise error–corrected significance level of p < 10⁻⁶. The overlaid binarized maps highlight regions functionally connected to symptom-related atrophy clusters for RBD, depression, and anxiety. Colours indicate network maps associated with RBD (green), depression (light blue), and anxiety (light orange). Images are displayed in neurological orientation (left = left, right = right; anterior = top, posterior = bottom).

**Supplementary Figure 2. The figure shows the coordinate-based connectivity networks for anxiety in Parkinson’s disease.** Connectivity maps were generated using seed-based functional connectivity T-maps from a large normative resting-state connectome (n=1000 healthy controls), and each map was thresholded at T ≥ 7, corresponding to a family-wise error–corrected significance level of p < 10⁻⁶. The resulting binarized maps represent brain regions functionally connected to the Voxel-Based Morphometry-derived clusters significantly associated with each clinical measure. Images are displayed in neurological orientation (left = left, right = right; anterior = top, posterior = bottom).

**Supplementary Figure 3. The figure shows the coordinate-based connectivity networks for depression in Parkinson’s disease.** Connectivity maps were generated using seed-based functional connectivity T-maps from a large normative resting-state connectome (n=1000 healthy controls), and each map was thresholded at T ≥ 7, corresponding to a family-wise error–corrected significance level of p < 10⁻⁶. The resulting binarized maps represent brain regions functionally connected to the Voxel-Based Morphometry-derived clusters significantly associated with each clinical measure. Images are displayed in neurological orientation (left = left, right = right; anterior = top, posterior = bottom).

**Supplementary Figure 4. The figure shows the coordinate-based connectivity networks for REM Sleep Behaviour Disorder (RBD) in PD.** Connectivity maps were generated using seed-based functional connectivity T-maps from a large normative resting-state connectome (n=1000 healthy controls), and each map was thresholded at T ≥ 7, corresponding to a family-wise error–corrected significance level of p < 10⁻⁶. The resulting binarized maps represent brain regions functionally connected to the Voxel-Based Morphometry-derived clusters significantly associated with each clinical measure. Images are displayed in neurological orientation (left = left, right = right; anterior = top, posterior = bottom).

**Supplementary Figure 5. Fold-enrichment analysis of the spatial overlap between symptom-related networks and canonical Yeo networks.** Heatmaps display the ratio of observed to expected overlap relative to random binary masks matched for voxel count. Values greater than 1 indicate higher overlap than expected by chance. Asterisks indicate statistically significant enrichment after false discovery rate (FDR) correction (q < 0.05) based on permutation testing with 10,000 iterations. The results should be interpreted alongside spatial similarity measures (Dice coefficients) and statistical significance, as described in the main text. Abbreviations: REM Sleep Behaviour Disorder = RBD.

**Supplementary Figure 6. Visual comparison between non-motor symptom–related networks in Parkinson’s disease patients and large-scale attention networks.** **A** Dorsal attention network (DAN) derived from the 7-network Yeo atlas. **B** Ventral attention network (VAN) derived from the 7-network Yeo atlas. **C** Functional network associated with REM Sleep Behaviour Disorder (RBD), obtained through coordinate-based network mapping of Voxel-Based Morphometry-derived regions. **D** Functional network associated with depression, obtained through coordinate-based network mapping of VBM-derived regions. **E** Functional network associated with anxiety, obtained through coordinate-based network mapping of VBM-derived regions. Connectivity maps were generated using seed-based functional connectivity T-maps from a large normative resting-state connectome (n=1000 healthy controls) and thresholded at T ≥ 7 (p < 10⁻⁶, family-wise error corrected). All networks are displayed on matched axial slices to facilitate direct visual comparison.
